# High expression of WISP1 in colon cancer is associated with apoptosis, invasion and poor prognosis

**DOI:** 10.18632/oncotarget.10486

**Published:** 2016-07-08

**Authors:** Jianghong Wu, Ziwen Long, Hong Cai, Chunyan Du, Xiaowen Liu, Shengjia Yu, Yanong Wang

**Affiliations:** ^1^ Department of Gastric Cancer and Soft Tissue Sarcoma, Fudan University Shanghai Cancer Center, Shanghai 200032, China; ^2^ Department of Oncology, Shanghai Medical College, Fudan University, Shanghai 200032, China

**Keywords:** WISP1, colon cancer, apoptosis, invasion, poor prognosis

## Abstract

Colon cancer (CC) likes many epithelial-derived cancers, resulting from a complex tumorigenic process. However, the exactly mechanisms of development and progression of CC are still unknown. In this study, integrated analysis in the GSE33113 and Fudan University Shanghai Cancer Center Hospital datasets revealed that WISP1 expression was significantly increased in CC cases, positivity correlated with the advanced pathologic stage and a poor prognosis was more likely in CC patients with higher levels of WISP1. Downregulation of WISP1 inhibited cell proliferation and invasion through increasing apoptosis and blocking cell cycle at G1 phase in CC LOVO and RKO cells. Besides, Gene set enrichment analysis (GSEA) revealed that relative genes involved in the Cell adhesion molecules and Cytokine-cytokine receptor interaction pathways were enriched in WISP1-higher expression patients. Western blot analysis showed that Cell adhesion molecules pathway associated genes (ICAM- 1, VCAM-1, SDC2 and CDH2) and Cytokine-cytokine receptor interaction pathway associated genes (VEGFC, CCL18, CXCR4 and TGFBR1) were also modulated by WISP1 downregulation. Then, we found that the protein β-catenin was identified as a binding partner of WISP1 and mediated the functions of WISP1 through promoting cell proliferation and invasion in LOVO and RKO cells. Further *in vivo* tumor formation study in nude mice indicated that inhibition of WISP1 delayed the progress of tumor formation and inhibited PCNA expression. These results indicate that WISP1 could act as an oncogene and may serve as a promising therapeutic strategy for colon cancer.

## INTRODUCTION

Colon cancer (CC) is the sixth most common cancer type and the fifth leading cause of cancer-related deaths in China [1]. Since dietary characteristics have changed in recent years, the mortality of colon cancer has been estimated 608,000 people each year in the world [2]. Meanwhile, the incidence of CC is increasing faster in recent years with estimated 274,841 cases and the mortality of that is 132,110 cases accounted for 48% of all the CC cases in China [1]. Although early screening, diagnosis and development of surgical resection, radiation therapy, chemotherapy and immunotherapy for CC, the survival rates of CC patients were almost not improved during the past decade. Importantly, the CC patients with metastasis have even lower survival rate compared with the patients in the absent of metastasis. Therefore, effective approaches for the treatment of CC patients with metastasis and for the decrease of recurrence are significantly important. Recently, many studies investigated whether there are any possible genes that associate with prognosis and tumorigenesis of CC, and WNT1 inducible signalling pathway protein 1, WISP1, has emerged as possible markers for human diseases including injuries and cancers [3].

WNT1 inducible signaling pathway protein 1 (WISP1, as known as CCN4) is a member of the CCN family as a whole appear to regulate proliferation, apoptosis, adhesion, migration and extracellular matrix production [3]. WISP1 has been originally implicated as a downstream target of WNT1 and β-catenin, and plays a role in regulating progresses of chondrocytic differentiation and tumorigenesis in cancer [4]. Furthermore, the expression of WISP1 was regulated by cellular processes that modulate the WNT1 and β-catenin signaling pathway [5]. It was reported that chronic alcohol feeding induces WNT1 and β-catenin signaling in alcohol-induced liver disease rat model and followed by WISP1 upregulation, resulting in promoting hepatic cell proliferation and tumorigenesis [6]. As its previous identification, WISP1 has been found in several cancers, such as prostate cancer [7], lung cancer [8], breast cancer [9], esophageal cancer [10] and colon cancer [11]. Previous studies showed that WISP1 was upregulated by WNT1 transformed mammary epithelial cells and subsequently conferred oncogenic characteristics in rat kidney cells, including stimulation of cell growth and tumor formation *in vitro* and *in vivo* [12]. In contrast, in lung cancer cells WISP1 overexpression led to invasion, migration and metastasis inhibition [13].

Genomic copies and mRNA of WISP1 were significantly increased in colon cancer tissues and cell lines compared with corresponding normal colorectal samples [14], suggesting that WISP1 may function in the development and progresses of CC, partially by accelerating cell proliferation in addition to promote cell cycle progression and inhibit cell apoptosis. However, the role of WISP1 in CC is controversial. For example, Khor et al. [15] links highly WISP1 expression to well-differentiated colon tumors, while Davies et al. shows higher WISP1 expression associated with poor differentiation, tumor invasion and poor prognosis outcome [16]. Despite the role of WISP1 in CC was investigated in recent years, the molecular mechanism of how WISP1 affects CC progression is not clear.

In the present study, we describe our study in helping understanding the functions of WISP1 in CC cell cycle, apoptosis and invasion. Bioinformatics and clinical characteristics analysis showed that WISP1 overexpressed in CC tissues and highly levels of WISP1 were associated with poor survival time and advanced pathological grade. Then we examined the biological functions of WISP1 in CC cell lines and found that WISP1 involved in multiple cellular progresses including cell proliferation, cell cycle, apoptosis, invasion, adhesion and cytokine-cytokine receptor interaction. And the protein β-catenin was identified as a binding partner of WISP1 and mediated the functions of WISP1. At last, *in vivo* tumor formation experiment showed that downregulation of WISP1 remarkably inhibited the tumor growth. These data suggest that WISP1 is an oncogene and a potential target for CC treatment.

## RESULTS

### Upregulation of WISP1 associates with poor survival of CC patients

**Figure 1:**
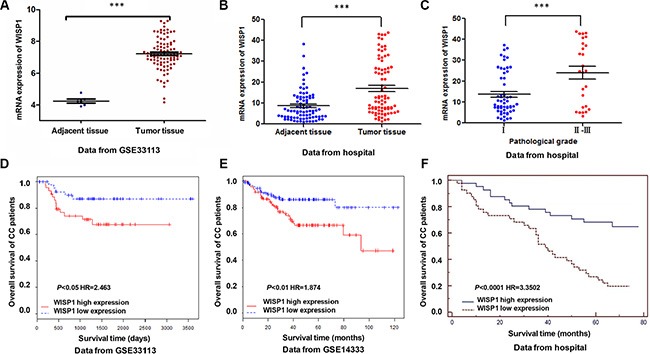
Correlation between WISP1 expression and survival time of patients with CC (**A**, **B**) Analysis of WISP1 expression level in CC samples by bioinformatics analysis in GSE33113 and Fudan University Shanghai Cancer Center Hospital datasets. (**C**) Statistical analysis on the pathological stage of CC patients in WISP1 expression from Fudan University Shanghai Cancer Center hospital. (**D**–**F**). Effect of the expression level of WISP1 on the overall survival of patients with CC in GSE33113, GSE14333 and Fudan University Shanghai Cancer Center Hospital datasets. The cut-off level was set at the median value of the WISP1 expression levels in CC patients. The WISP1-higher expression tumors have a poor prognosis compared to the WISP1-lower expression tumors. ***P < 0.001.

WISP1 expression was significantly increased in CC tissues when compared with the adjacent tissues of patients from GEO dataset (Access id: GSE33113) and Fudan University Shanghai Cancer Center Hospital independent dataset (Figure 1A and 1B). Then, we investigated the correlation between WISP1 expression and clinicopathological features of the CC patients in Fudan University Shanghai Cancer Center Hospital. We detected the expression level of WISP1 of 82 CC patients' tissues divided into two group using WISP1 median value. Chi-square test indicated that evaluation of WISP1 expression in 82 CC patients with different clinicopathological features revealed that the WISP1 expression was positively correlated with the advanced pathological stage (Figure 1C). However, WISP1 expression was not correlated with gender, age, tumor volume and clinical stage (Table 1).

**Table 1:** Correlation of the expression of WISP1 with clinicopathologic features

Clinicopathologic features	*n* (%)	Relative expression of WISP1	*P*-value
Gender			0.663
Male	43 (52)	17.62	
Female	39 (48)	16.37	
Age			0.416
< 60	26 (32)	0.740	
> 60	56 (68)	0.744	
Tumor volume (cm^3^)			0.198
< 10	16 (20)	0.696	
> 10	66 (80)	0.790	
Clinical stage			0.934
I/II	45 (55)	16.92	
III/IV	37 (45)	17.16	

The survival time of 82 CC patients showed that lower-WISP1-expressing patients notably lived longer than higher-WISP1-expressing patients from GEO dataset (Access id: GSE33113, GSE14333) (Figure 1D and 1E). We also analyzed data of CC patients in Fudan University Shanghai Cancer Center Hospital, which is similar to the results of GEO dataset (Figure 1F). These results all supported our findings that WISP1 expression was upregulated in CC patients which correlated with poor CC patient survival.

### Downregulation of WISP1 represses CC cell proliferation

**Figure 2:**
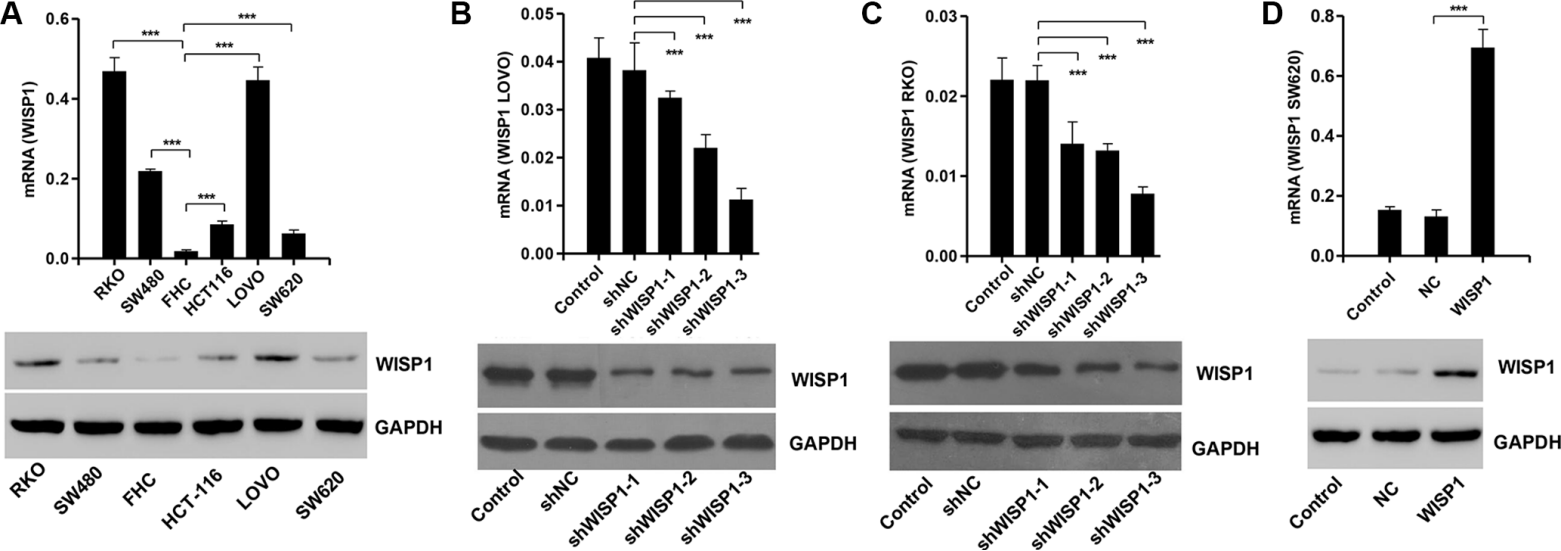
WISP1 overexpression and knockdown by shRNA in CC cell lines (**A**) Real-time PCR and Western blot analysis identified significant increase in WISP1 expression in five CC cells and normal epithelial colon cells. (**B**, **C**) Real-time PCR and Western blot analysis identified significant decrease in WISP1 expression in LOVO and RKO cells treated with WISP1 shRNAs. (**D**) Real-time PCR and Western blot analysis identified significant increase in WISP1 expression in SW620 cells treated with pLVX-AcGFP-C1-WISP1 expressing vector (WISP1). shNC: pLVX-AcGFP-C1-scramble shRNA negative control. ***P < 0.001.

**Figure 3:**
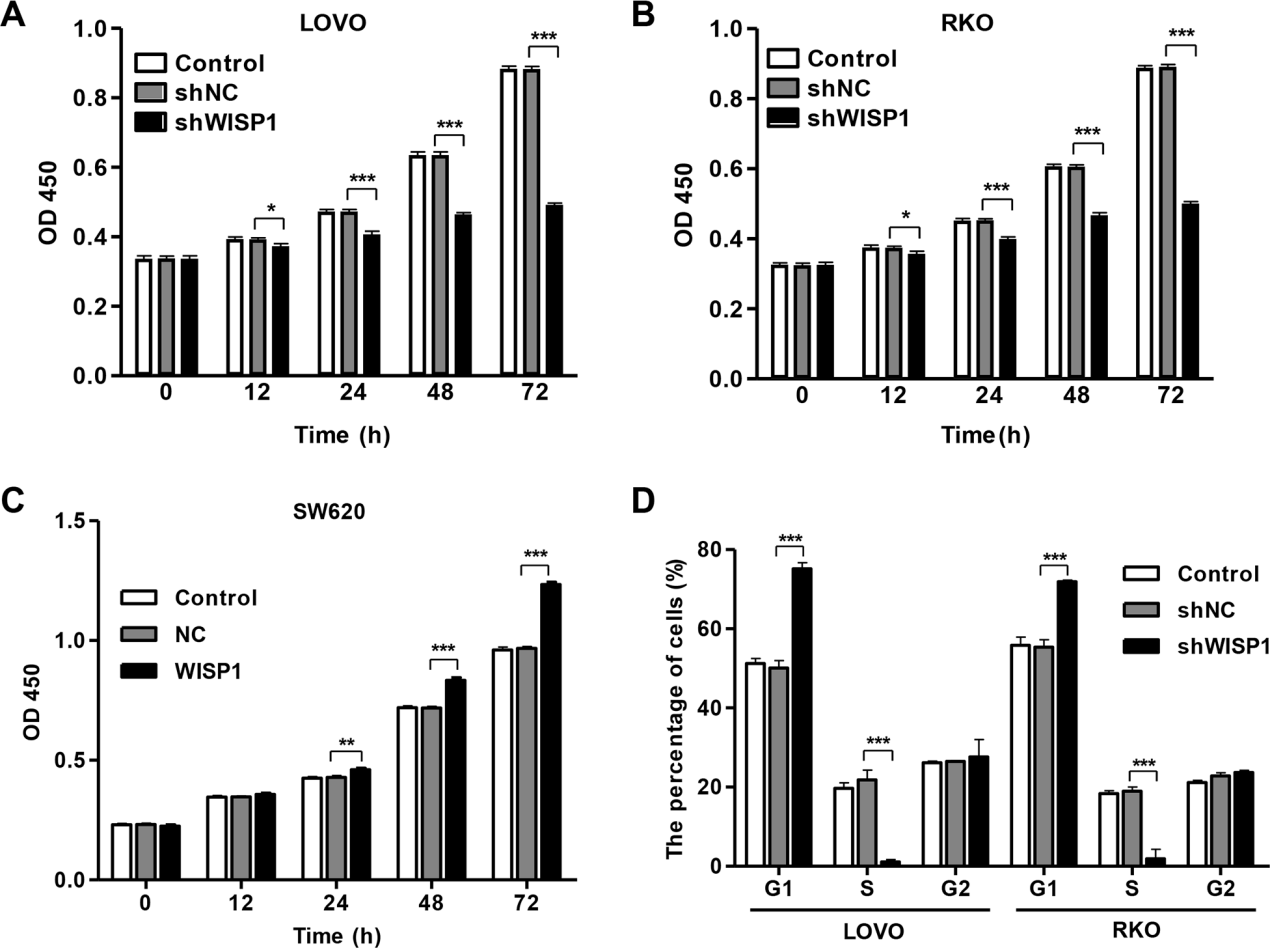
WISP1 shRNA inhibits cell proliferation by arresting cells at G1 phase LOVO, RKO and SW620 cells were infected with pLVX-AcGFP-C1-shWISP1 (shWISP1) or pLVX-AcGFP-C1-WISP1 expressing vector (WISP1) after 48 h. (**A**–**C**). Cells proliferation was detected by CCK-8 assay in LOVO, RKO and SW620 cells. (**D**) Cell cycle profile was analyzed using flow cytometry in LOVO and RKO cells. shNC: pLVX-AcGFP-C1-scramble shRNA negative control. NC: black pLVX-AcGFP-C1 negative control. *P < 0.05, ***P < 0.001.

Having documented upregulation of WISP1 associates with poor prognosis of CC patients, we wonder how WISP1 affects CC cell biological behavior. We analyzed WISP1 expression in 5 CC cell lines, RKO, SW480, SW620, HCT-116 and LOVO, and normal epithelial colon cells (FHC) by Real-time PCR and Western blot (Figure 2A). WISP1 was expressed in higher level in five CC cell lines specifically in LOVO and RKO cell lines compared with the FHC cell lines, while WISP1 was expressed in lower level in SW620 cells. Then, pLVX-AcGFP-C1-shWISP1 (shWISP1) were stably infected into LOVO and RKO cells (Figure 2B and 2C) and pLVX-AcGFP-C1-WISP1 (WISP1) expressing vector was stably infected into SW620 cells (Figure 2D). The highest infection efficiency was observed in shWISP1-3 infected cells and thus shWISP1-3 was used in the following experiments. After infection, cell proliferation was analyzed in higher-WISP1 expression cell lines LOVO and RKO and lower-expression cell line SW620 by using CCK-8 assay. As shown in Figure 3A and 3B, downregulation of WISP1 through infection of shWISP1 into LOVO and RKO cells induced great inhibition on cell proliferation compared with pLVX-AcGFP-C1-scramble shRNA negative control (shNC) in both cell lines as early as 12 h later. While, overexpression of WISP1 in lower-WISP1 expression cell line SW620 increased cell proliferation compared with black pLVX-AcGFP-C1 negative control (NC) (Figure 3C).

To further validate the cell proliferation inhibition of anti-WISP1, cell cycle was analyzed in LOVO and RKO cells. Cell cycle analysis showed that downregulation of WISP1 notably increased the rate of G1 phase cells and reduced S phase cell population in both cell lines (Figure 3D). These results indicated that downregulation of WISP1 in CC cells may inhibit cell proliferation by arresting cell cycle progression in G1 phase.

### Downregulation of WISP1 induces cell apoptosis and inhibits invasion in CC cells

**Figure 4:**
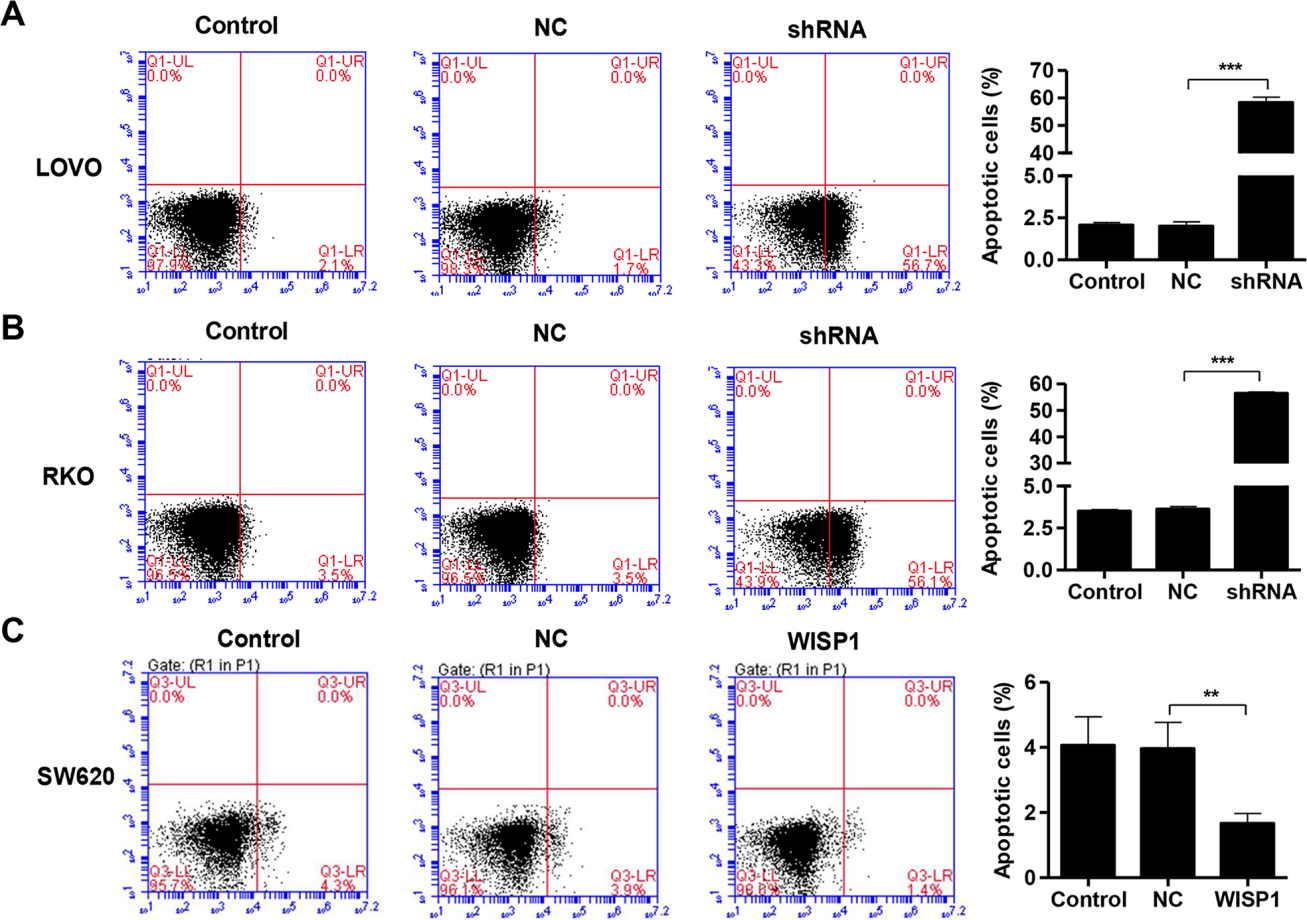
WISP1 shRNA promotes cell apoptosis (**A**–**C**). LOVO, RKO and SW620 cells treatment with pLVX-AcGFP-C1-shWISP1 (shWISP1) or pLVX-AcGFP-C1-WISP1 expressing vector (WISP1) were stained with annexin V-fluorescein and apoptosis rates was analyzed using flow cytometry. shNC: pLVX-AcGFP-C1-scramble shRNA negative control. NC: black pLVX-AcGFP-C1 negative control. ***P < 0.001.

**Figure 5:**
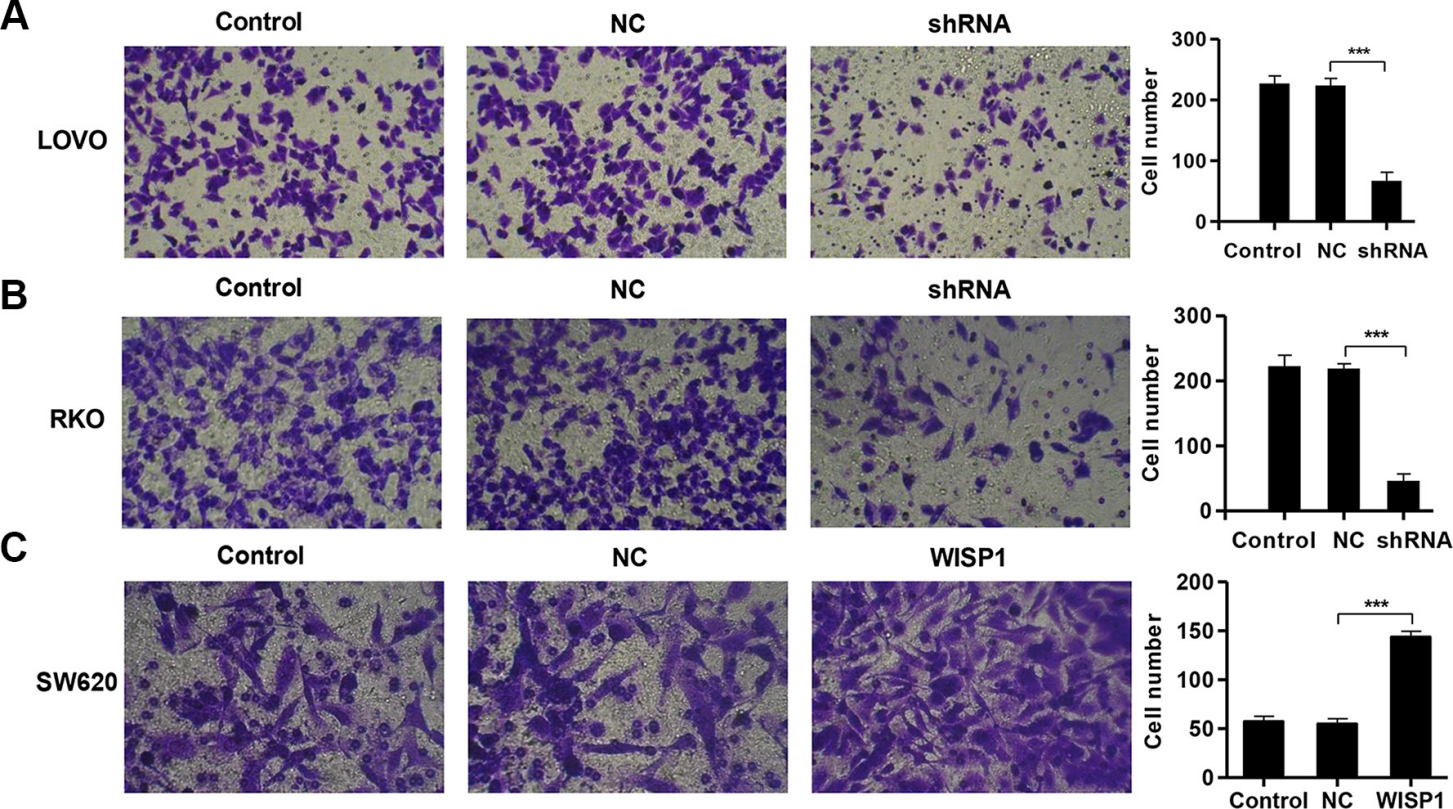
WISP1 shRNA inhibits invasion (**A**–**C**). LOVO, RKO and SW620 cells treatment with pLVX-AcGFP-C1-shWISP1 (shWISP1) or pLVX-AcGFP-C1-WISP1 expressing vector (WISP1) and invasion was determined by transwell assays. shNC: pLVX-AcGFP-C1-scramble shRNA negative control. NC: black pLVX-AcGFP-C1 negative control. ***P < 0.001.

Then, we evaluated the apoptotic function of WISP1 in LOVO and RKO cells by Annexin V-FITC/PI staining assay. As shown in Figure 4A and 4B, flow cytometry analysis revealed that downregulation of WISP1 in LOVO and RKO cells significantly induced cell apoptosis compared to shNC. While, overexpression of WISP1 in SW620 cells reduced cell apoptosis compared with NC (Figure 4C). Moreover, invasion abilities of LOVO and RKO cells infected shWISP1 were also examined by transwell assay after 48 h. The results showed that invasive cells were significantly decreased in shWISP1 treatment group compared with that in shNC (Figure 5A and 5B). While, for SW620 cells overexpression of WISP1 resulted in high level of cell invasion (Figure 5C). These data suggest that WISP1 may function as a tumor promoter in CC through inducing cell apoptosis and inhibiting cell invasion.

### WISP1 regulates cell adhesion molecules and cytokine-cytokine receptor interaction pathways

**Figure 6:**
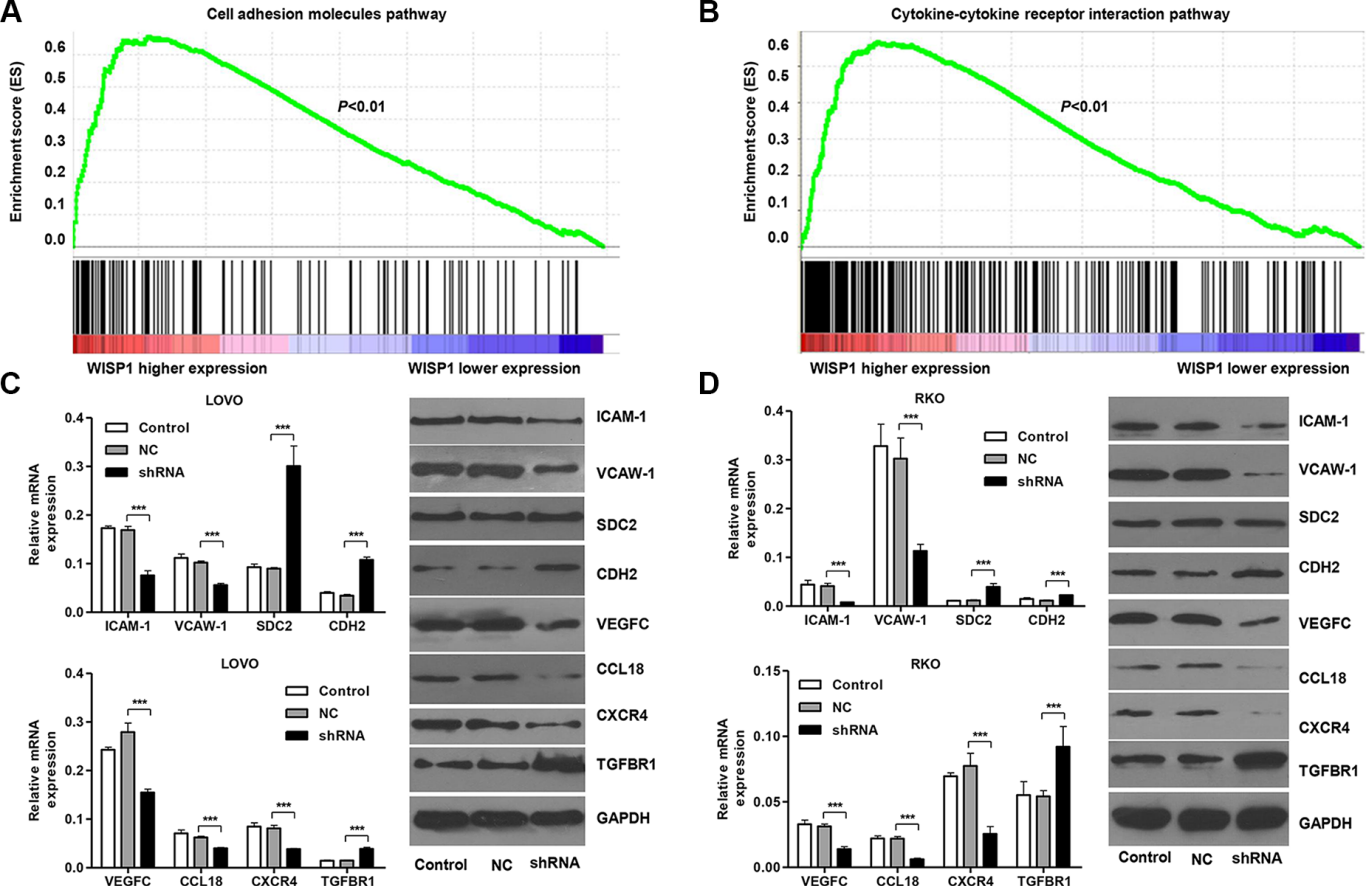
WISP1 regulates cell adhesion molecules and cytokine-cytokine receptor interaction pathways (**A**, **B**). Genes in the Cell adhesion molecules and Cytokine-cytokine receptor interaction pathways showed significant enrichment in WISP1 high versus WISP1 low tumors in CC patients. The top portion of the figure plots the enrichment scores (ES) for each gene, whereas the bottom portion of the plot shows the value of the ranking metric moving down the list of ranked genes. (**C**, **D**). Real-time PCR and Western blot analysis identified significant decrease in ICAM-1, VCAW-1, VEGFC, CCL18 and CXCR4, while increase in SDC2, CDH2 and TGFBR1 expression in LOVO and RKO cells infected with pLVX-AcGFP-C1-shWISP1 (shWISP1). shNC: pLVX-AcGFP-C1-scramble shRNA negative control. NC: black pLVX-AcGFP-C1 negative control. ***P < 0.001.

The exact pathways that WISP1 may regulate in CC remain unclear. To probe the WISP1-associated pathways on an unbiased basis, we performed GSEA using high throughput RNA-sequencing data of the CC cohort of KEGG dataset. Among all the predefined KEGG gene sets, the KEGG Cell adhesion molecules and Cytokine-cytokine receptor interaction pathways were identified with the strongest association with WISP1 expression. Enrichment plots of GSEA showed that the gene signatures of Cell adhesion molecules and Cytokine-cytokine receptor interaction pathways were more correlated with patients with WISP1-higher expression versus patients with WISP1-lower expression (Figure 6A and 6B). A positive ranking metric indicates that the gene is correlated with the CC phenotype.

Then, we investigate the effects of WISP1 on cell adhesion and Cytokine-cytokine receptor interaction in CC cells. Real-time PCR and Western blot analyses showed that alteration of WISP1 expression significantly changed the expression of the Cell adhesion molecules markers, ICAM-1, VCAM-1, SDC2, CDH2 and Cytokine-cytokine receptor interaction markers, VEGFC, CCL18, CXCR4, TGFBR1 in LOVO and RKO cells (Figure 6C and 6D). The data were consistent with the correlation of WISP1 with pathological features and GSEA analysis in CC.

### Association of WISP1 with β-catenin

**Figure 7:**
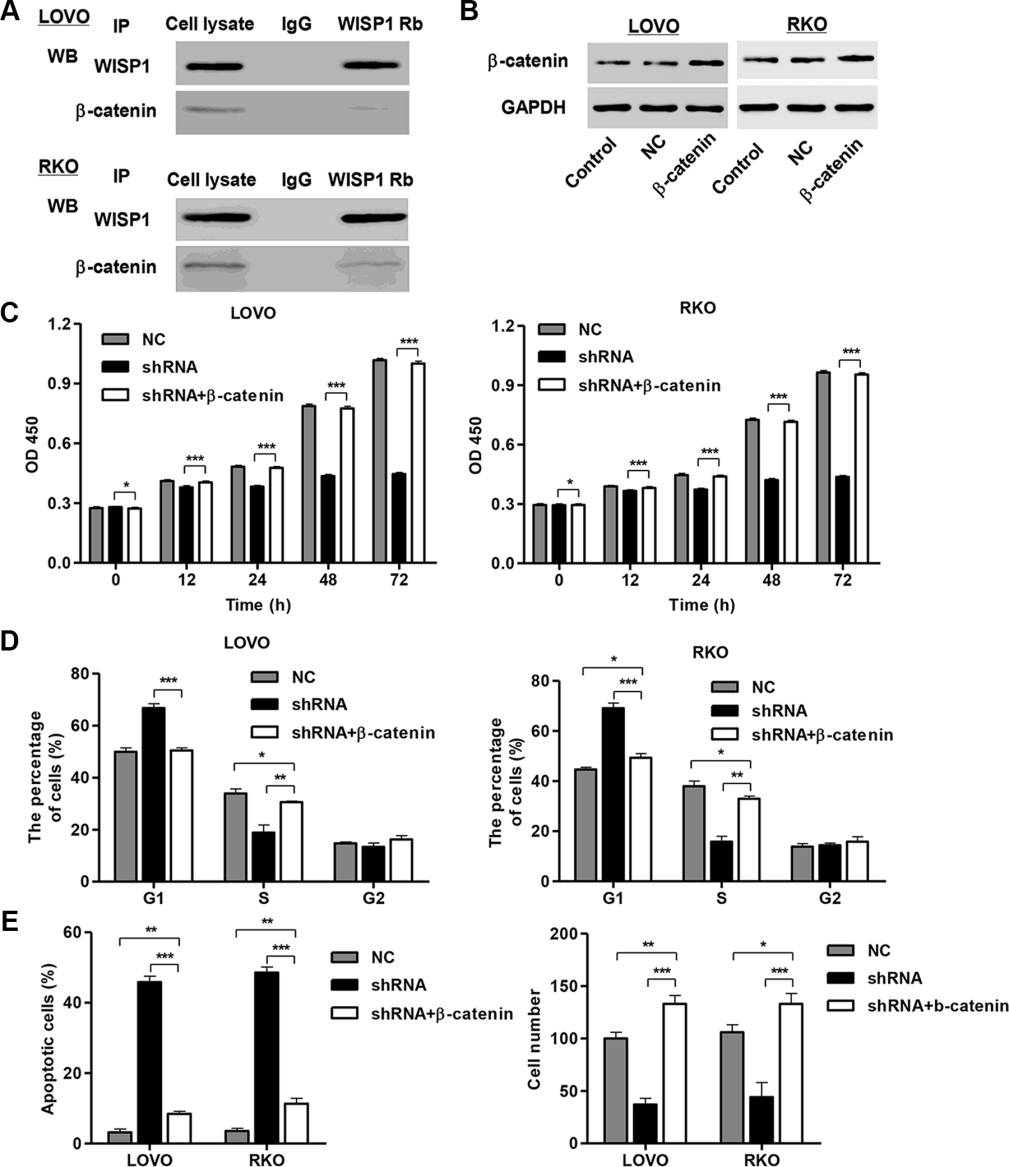
WISP1 binds to β-catenin *in vitro* (**A**) Co-immunoprecipitation showed that WISP1 interacts with β-catenin in the cell lines LOVO and RKO cells. (**B**) β-catenin overexpression in LOVO and RKO cells. (**C**, **D**). Cells proliferation was detected by CCK-8 assay and cell cycle profile was analyzed using flow cytometry in LOVO and RKO cells infected with pLVX-AcGFP-C1-shWISP1 (shWISP1) and pLKO.1-EGFP-β-catenin expressing vector (β-catenin). (**E**) Apoptosis rate was analyzed using flow cytometry and invasion was determined by transwell assays. NC: black pLKO.1-EGFP negative control. shNC: pLVX-AcGFP-C1-scramble shRNA negative control. *P < 0.05, **P < 0.01, ***P < 0.001.

To elucidate the underlying mechanisms by which WISP1 exerts its function in CC pathogenesis, we identified protein candidates that functionally associated with WISP1. As shown in Figure 7A, we explored the nature of the interaction between WISP1 and β-catenin by using Co-immunoprecipitiation in both LOVO and RKO cells. The result obtained from the Co-immunoprecipitiation experiments indicated that β-catenin directly interacts with WISP1. As β-catenin directly interacted with WISP1, we wonder whether β-catenin associated with the CC tumorigenesis. We thus performed proliferation, cell cycle, apoptosis and invasion assays in β-catenin overexpressed LOVO and RKO cells. As known in Figure 7B, β-catenin overexpressed LOVO and RKO cells showed highly expression of β-catenin. And, β-catenin overexpression significantly promoted cell proliferation (Figure 7C), cell cycle arrest at G1 phase (Figure 7D) and invasion and inhibited cell apoptosis (Figure 7E) compared with the corresponding control. These results suggested that β-catenin may mediate the functions of WISP1 in CC tumorigenesis.

### Downregulation of WISP1 suppresses tumor growth and metastasis of CC *in vivo*

**Figure 8:**
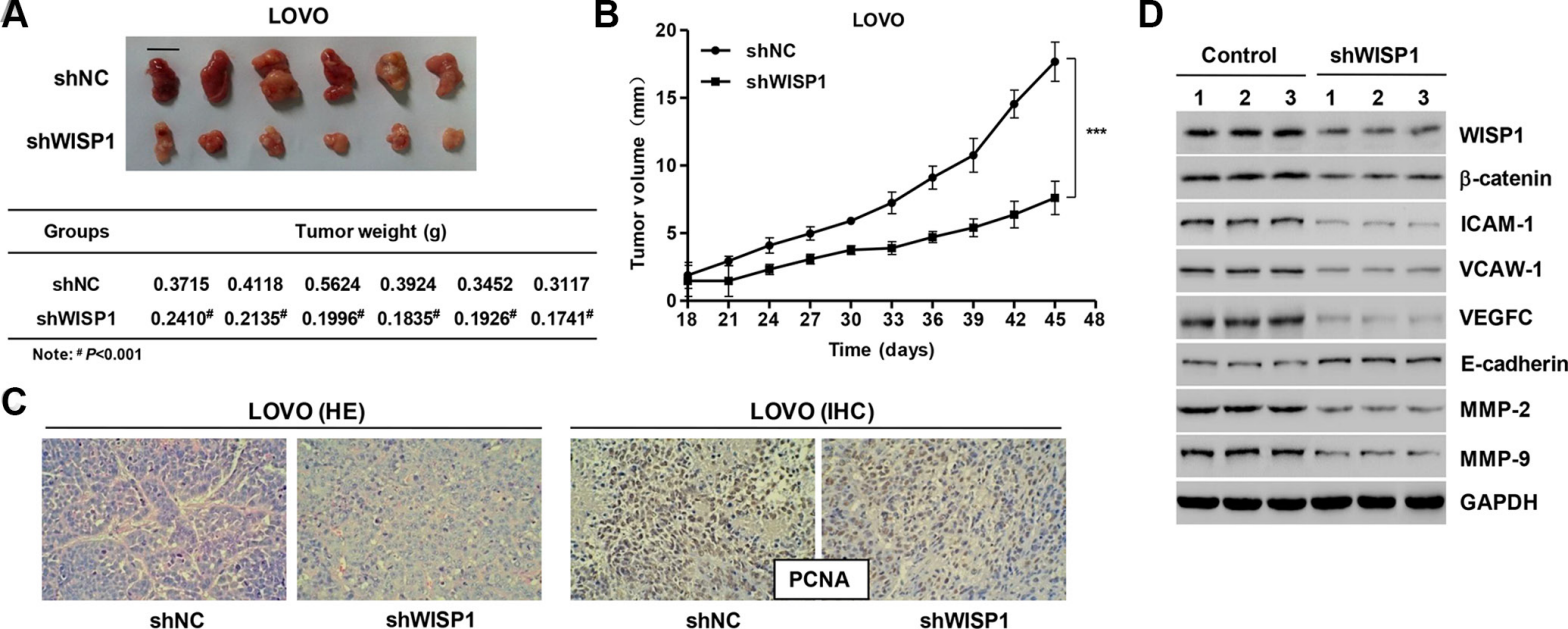
Knockdown of WISP1 in CC cells reduces tumor growth *in vivo* LOVO cells infected with pLVX-AcGFP-C1-scramble shRNA negative control (shNC) or pLVX-AcGFP-C1-shWISP1 (shWISP1) were subcutaneously injected in athymic nude mice. (**A**) At day 45, mice were sacrificed and tumors were weighted. (**B**) Tumor growth was evaluated for 45 days. (**C**) Histology and PCNA expression were detected by HE staining and immunohistochemistry (IHC) assays. (**D**) Western blot analysis identified significant decrease in WISP1, β-catenin, ICAM-1, VCAW-1, VEGFC, MMP-2 and MMP-9, while increase in E-cadherin expression in LOVO cells infected with pLVX-AcGFP-C1-shWISP1 (shWISP1). shNC: pLVX-AcGFP-C1-scramble shRNA negative control. Scale bar: 10 mm. ^#^*P* < 0.001, ***P < 0.001.

Next, we determined whether silence of WISP1 in CC cells could reduce tumor growth *in vivo*. LOVO cells infected with pLVX-AcGFP-C1-scramble shNRA negative control (shNC) and WISP1 shRNA (shWISP1) were subcutaneously injected in athymic nude mice respectively. After 45 days, tumor weight in mice with LOVO cells infected with shWISP1 was significant decrease compared to the shNC mice (Figure 8A). Tumor volumes were measured for 45 days. As shown in Figure 8B, tumor volume in WISP1 downregulated tumors in mice was significant decrease compared to the shNC mice whereas shNC tumors grew fast in mice. Additionally, the histology and immunohistochemistry (IHC) assays were also detected in mice with LOVO cells infected with shWISP1 and showed that the expression of PCNA was significantly decreased compared with shNC mice (Figure 8C). Western blot analysis showed that downregulation of WISP1 expression significantly decreased the expression of β-catenin and the metastasis-related proteins, including ICAM-1, VCAM-1, VEGFC, MMP-2 and MMP-9, and increased the E-cadherin expression *in vivo* (Figure 8D). These data suggested that downregulation of WISP1 in CC cells reduces tumor growth and represses tumor metastasis in nude mice.

## DISCUSSION

In the current study, we demonstrated the biological functions of WISP1 in CC. The clinical characteristics showed that WISP1 was upregulated in CC patients which was supported by the data from GSE33113 and Fudan University Shanghai Cancer Center Hospital. Highly level of WISP1 was associated with the advanced pathological stage and poor survival time. *In vitro* experiments demonstrated that downregulation of WISP1 in CC cells inhibited proliferation and invasion and induced cell cycle arrest and apoptosis. Cell adhesion molecules and Cytokine-cytokine receptor interaction pathways were also regulated in response to WISP1. Furthermore, the protein β-catenin was identified as a binding partner of WISP1 in CC cells. Therefore, WISP1 may function as a useful prognosis marker and potential target for CC treatment.

WISP1 is aberrantly expressed in a number of diseases, including injury and cancer [3]. WISP1 is expressed during cell injury such as inflammatory lung injury [17], cartilage injury [18] myocardial injury [19] and neuronal injury [20]. Increased expression of WISP1 during neuronal injury may have a significant correlation with enhanced cellular survival and WISP1 may serve as a therapeutic target against neurodegenerative disorders such as Parkinson's disease and Alzheimer's disease. However, the roles of WISP1 in different cancers were significant varieties. For example, the highly levels of WISP1 were found in breast [21], rectal [22] and esophageal cancer [23] with poor prognosis, but WISP1 expressed lower levels in melanoma with poor prognosis [24]. A similar discrepancy is seen when analyzing WISP1 expression in CC [15, 16]. Moreover, significant associations were found between WISP1 mRNA levels versus stage, tumor size, lymph node in primary breast cancer [21]. We examined the relationship between WISP1 expression and clinical characterstics especially patient's prognosis in GSE33113, GSE14333 and Fudan University Shanghai Cancer Center Hospital data. We found highly expression of WISP1 associated with advanced pathological grade and poor survival time. These data suggest the oncogenic role of WISP1 in CC.

Then, we investigated the role of WISP1 in CC LOVO, RKO and SW620 cell lines by measured cell proliferation, cell cycle, apoptosis and invasion. Downregulation of WISP1 significantly inhibited cell proliferation, G1/S cell cycle transition and invasion, and induced apoptosis. While, overexpression of WISP1 promoted cell proliferation and invasion and inhibited apoptosis. Further *in vivo* tumor formation study in nude mice indicated that WISP1 downregulation in CC cells suppressed tumor formation. Many reports supported a function of WISP1 in cancer cell proliferation, apoptosis, invasion *in vitro* and tumor growth *in vivo*. Inhibition of WISP1 repressed the tumor growth and invasion of prostate cancer *in vivo* and *in vitro* [7]. Su et al. reported that activation of AKT and Bcl-XL, and inhibition of cytochrome c release were involved in WISP1 protected cells from p53-dependent apoptosis [25]. In contrast, overexpression of WISP1 inhibited proliferation and cell invasion in melanoma [24] and lung cancer cells [13].

Moreover, it has been shown that cell adhesion molecules and cytokine-cytokine receptor interaction are associated with the progress of several cancers such as breast [26, 27] and prostate [28]. In this study, Cell adhesion molecules and Cytokine-cytokine receptor interaction pathways were identified with the strongest association with WISP1 expression in patients from KEGG data. According to the Western blot analysis *in vitro*, Cell adhesion molecules pathway associated gene expressions were significantly decreased (ICAM-1 and VCAM-1) and increased (SDC2 and CDH2) by WISP1 downregulation in LOVO and RKO cells. Meanwhile, Cytokine-cytokine receptor interaction pathway associated gene expression were also decreased (VEGFC, CCL18 and CXCR4) and increased (TGFBR1) by WISP1 downregulation in LOVO and RKO cells. These results indicated that the effect of WISP1 in CC progress was involved in the correlation with Cell adhesion molecules and Cytokine-cytokine receptor interaction pathways. Importantly, downregulation of WISP1 *in vivo* also induced decrease in expression of ICAM-1, VCAM-1, VEGFC, MMP-2, MMP-9 and increase in E-cadherin, suggesting that WISP1 downregulation not only inhibited tumor growth but also suppressed tumor metastasis *in vivo*. Indeed, ICAM-1 and VCAM-1 expression correlates with increased metastasis and determines malignant potential of cancer [29. However, overexpression of SDC2 and CDH2 enhanced migration and invasion of human CC Caco-2 and SW620 cells [30, 31]. Cytokine and cytokine receptor play an important role in the progression of cancers. They are involved in tumor growth, angiogenesis and metastasis. The expression of cytokine and their receptors is altered in many malignancies and subsequently leads to aberrant cytokine receptor signaling. For example, CXCR4 is overexpressed in ovarian and B-cell chronic lymphocytic leukemia and is also involved in their proliferation, apoptosis and metastasis [32, 33]. The knockdown of CXCR4 in CC cells leads to a decrease in cell migration [34]. CCL18 enhances proliferation and metastasis of ovarian cancer cell lines [35]. Furthermore, downregulation of VEGFC expression in CC cells decelerates tumor growth and inhibits metastasis [36]. In contrast, microRNA-140-5p suppresses tumor growth and metastasis by targeting TGFBR1 in hepatocellular carcinoma [37]. Various proteases are involved in cancer progression and metastasis. In particular, MMP-2 and MMP-9 have been implicated to play a role in colon cancer progression and metastasis in animal models and patients [38]. Taken together, WISPI involved in CC initiation and malignancy may via regulating these cell adhesion molecules and cytokine-cytokine receptors in CC cells.

How exactly WISP1 regulates cellular function and through which receptors it transmits signals is unknown. Recent studies have demonstrated the functional interaction of WISP1 with decorin and biglycan, which present in the extracellular matrix of connective tissue [39]. Another binding partner of WISP1 is α5β1 integrin, and WISP1 overexpression increases α5 expression in bone marrow stromal cells [40]. In this study, we revealed that WISP1 directly bound to β-catenin in both LOVO and RKO cells, suggesting that implicate signaling through β-catenin pathway as a critical downstream mechanism by which WISP1 may regulate changes in the cell proliferation, cell cycle, apoptosis and invasion. Hyperactivation of Wnt/β-catenin signaling is one of the earliest events in the pathogenesis of colon cancer and is implicated in the metabolic diseases [41]. The deleted in liver cancer-1 (DLC-1) overexpression inhibited CC cell proliferation, colony formation and invasion, and induced cell cycle arrest at the G1 phase with subsequent apoptosis, possibly through the regulation of the Wnt/β-catenin signaling pathway [42].

In summary, our study provides for the first time that WISP1 plays an important role in CC cell proliferation, apoptosis, invasion, adhesion and cytokine-cytokine receptor interaction, and WISP1 may regulate these biological progresses through directly bound to β-catenin. As the relationship between WISP1 and clinical characteristics, inhibition of WISP1 in CC patients may serve a therapeutic strategy. Therefore, the roles of WISP1 in CC tumorigensis need further investigation.

## MATERIALS AND METHODS

### Patients and tissue samples

Tumor and normal colon specimens were obtained from 82 CC patients who underwent surgery at Fudan University Shanghai Cancer Center Hospital from Jun 2008 to Apr 2013. The patients' clinical characteristics such as age, gender, tumor size, clinical stage and survival rate were collected for statistical analysis. The study protocol was approved by the ethics committee of Fudan University Shanghai Cancer Center Hospital. Written informed consents were obtained from all participants in this study.

### Cell culture

Human CC cell lines RKO, SW480, SW620, HCT-116 and LOVO, and normal epithelial colon cells (FHC) were obtained from the Shanghai Cell Bank, Chinese Academy of Sciences. RKO, SW480, HCT-116 LOVO and FHC cells were cultured in DMEM (Biowest) supplemented with 10% fetal bovine serum (Gibco), 1% penicillin and streptomycin (Gibco). SW620 cells were grown in RPMI 1640 supplemented with 10% fetal bovine serum, 1% penicillin and streptomycin. Cells were incubated in a humidified atmosphere at 37°C with 5% CO_2_.

### Lentivirus transduction

pLVX-AcGFP-C1 and pLKO.1-EGFP were purchased from Addgene. WISP1 shRNA and plasmid containing full length of WISP1 or β-catenin were purchased from Funeng Gene co., LTD., Guangzhou. Oligonucleotides encoding shRNAs directed against human WISP1 (shWISP1-1-F: CCAGGUCCUAUGGAUUAAUTT, shWISP1-1-R: AUUA AUCCAUAGGACCUGGTT; shWISP1-2-F: CCUACGAC CAUGGACUUUATT, shWISP1-2-R: UAAAGUCCAUG GUCGUAGGTT; shWISP1-3-F: GCUCCUAUCAACCCA AGUATT, shWISP1-3-R: UACUUGGGUUGAUAGGAGC TT) were purchased from Invitrogen, USA. The WISP1 shRNA or coding sequence was cloned into the pLVX-AcGFP-C1 lentiviral vector. The recombinant lentivirus pLVX-AcGFP-C1-scramble shRNA (shNC) and black pLVX-AcGFP-C1 were used as the negative control (NC). The β-catenin coding sequence was cloned into the pLKO.1-EGFP lentiviral vector. A blank pLKO.1-EGFP lentiviral vector was used as negative control (NC). Then 293T cells were seeded in 60 mm dishes and after 24 h were co-transfected with 2 μg of the plasmid vector, 1μg pLKO.1-EGFP-β-catenin/pLVX-AcGFP-C1-shWISP1/pLVX-AcGFP-C1-WISP1, 0.1 μg VSV-G and 0.9 μg pol/gag by using lipofectamine 2000 (Invitrogen Life Technologies) according to the manufacturer's instruction. The recombinant lentivirus vector were collected 48 h after transfection and used to infect LOVO, RKO and SW620 cells at an multiplicity of infection (MOI) of 20 in the presence of 8 μg/ml polybrene (Sigma-Aldrich, St. Louis, MO, USA), respectively.

### Cell proliferation assay

Cell proliferation was assessed by Cell Counting Kit (CCK)-8 kit (Tongren) as previously described [43]. Briefly, LOVO, RKO and SW620 cells (4 × 10^3^) were seeded in each 96-well plates and further incubated for 0 h, 12 h, 24 h, 48 h and 72 h, respectively. At the indicated time points, CCK-8 solution (10 μl in 100 μl DMEM or RPMI 1640) was added to each well and incubated for 1 h at 37°C. The optical density (OD) 450 nm values in each well were determined by a microplate reader.

### Cell cycle and apoptosis assay

The percentages of cells in the different phases of cell cycle were evaluated by determining the DNA content after propidium iodide (PI) staining (BD Biosciences). Briefly, LOVO and RKO cells (4 × 10^3^) were harvested 48 h and incubated in PBS containing RNase (1 mg/mL) for 10 min at room temperature. Finally, samples were stained with propidium iodide (1 mg/mL) for 30 min at 4°C. Data acquisition was done by flow cytometry (FACSCalibur, BD Biosciences) using Cell Quest software. Annexin V/PI staining (BD Biosciences) and flow cytometry analysis were performed according to the manufacturer's instruction. Briefly, LOVO, RKO and SW620 cells were incubated with Annexin V FITC and PI prior to analysis using a flow cytometer.

### Cell invasion assay

Invasion assays were performed using Transwell chamber (Greiner Bio-One) coated with Matrigel (BD Biosciences) as described in the manufacturer's protocol. Briefly, LOVO, RKO and SW620 cells were serumstarved for 24 h and subsequently 1 × 10^5^ of cells in 500 μl DMEM or RPMI 1640 were plated in the top chamber of the insert precoated with Matrigel. Cell culture medium, supplemented with 10% FBS, was added into the lower well of the chamber. After 48 h of incubation, the cells on the upper well and the membranes coated with Matrigel were swabbed with a Q-tip. Then cells were fixed with 4% paraformaldehyde (Gibco) for 10 min and stained with Giemsa (Gibco) for 30 min. Cells were photographed and counted under microscopy in random 10 fields with magnification of ×200.

### Reverse transcription and Real-time PCR

Total RNA was isolated using Trizol reagent (Invitrogen Life Technologies). Reverse transcription reactions were performed as described [44]. Briefly, cDNA was reverse transcribed from RNA using a cDNA synthesis kit (Thermo Fisher Scientific, Inc., Rockford, IL, USA). The cDNA synthesis conditions were as follows: 37°C for 60 min, followed by 85°C for 5 min and 4°C for 5 min. Real-time PCR was performed using a standard SYBR Green PCR kit protocol on ABI7300 (Applied Biosystem) thermal cycler. The Real-time-PCR cycling conditions were as follows: 95°C for 10 min, followed by 40 cycles at 95°C for 15 sec and 60°C for 45 sec and a final extension step of 95°C for 15 sec, 60°C for 1 min, 95°C for 15 sec and 60°C for 15 sec. The primers sequences (sense/antisense) used were list in Table 2. The relative mRNA expressions of indicated genes were calculated using the ΔΔ*C*t method.

**Table 2:** Primes sequences used in this study

Gene	Sequences
WISP1-forward	5′-GAAGCAGTCAGCCCTTATG-3′
WISP1-reverse	5′-CTTGGGTGTAGTCCAGAAC-3′
ICAM-1-forward	5′-GTTGTTGGGCATAGAGAC-3′
ICAM-1-reverse	5′-CAGGGCAGTTTGAATAGC-3′
VCAM-1-forward	5′-TGGGAACGAACACTCTTAC-3′
VCAM-1-reverse	5′-CAGCAACTGAACACTTGAC-3′
SDC2-forward	5′-AAACCACGACGCTGAATATAC-3′
SDC2-reverse	5′-AATAACTCCACCAGCAATGAC-3′
CDH2-forward	5′-CATCATCCTGCTTATCCTTG-3′
CDH2-reverse	5′-AAGTCATAGTCCTGGTCTTC-3′
VEGFC-forward	5′-CACTTGCTGGGCTTCTTCTC-3′
VEGFC-reverse	5′-CACTGGACACAGACCGTAAC-3′
CCL18-forward	5′-TAAGAGTCCCATCTGCTATGC-3′
CCL18-reverse	5′-GCACAATGTCTGCTGAGAAAG-3′
CXCR4-forward	5′-CCTGTCCTGCTATTGCATTATC-3′
CXCR4-reverse	5′-TGCACAGTGTTCTCAAACTC-3′
TGFBR1-forward	5′-TGTGAAGCCTTGAGAGTAATG-3′
TGFBR1-reverse	5′-GTTGACTGAGTTGCGATAATG-3′
GAPDH-forward	5′-CACCCACTCCTCCACCTTTG-3′
GAPDH-reverse	5′-CCACCACCCTGTTGCTGTAG-3′

### Western blot

Treated and untreated colon cancer cells were centrifuged at 13,000 × *g* for 10 min at 4°C and the supernatant (20–30 μg of protein) was run on 10% SDS-PAGE gel and transferred electrophoretically to a polyvinylidene fluoride membrane (Millipore, Shanghai, China). The blots were blocked with 5% skim milk, followed by incubation with antibodies against ICAM-1, VCAM-1, VEGFC, CXCR4, TGFBR1, E-cadherin, MMP-2 and MMP-9 were purchased from Abcam (Cambridge, MA, USA); antibodies against SDC2, CDH2 and CCL18 were purchased from Santa Cruz Biotechnology (Dallas, TX, USA); antibody against GAPDH was purchased from Cell Signaling Technology (Danvers, MA, USA). Blots were then incubated with goat anti-mouse or anti-rabbit secondary antibody (Beyotime, Shanghai, China) and visualized using enhanced chemiluminescence (Thermo Scientific).

### Co-immunoprecipitation (Co-IP)

Co-immunoprecipitation was performed as described previously [45]. Both the input and IP samples were analyzed by Western blot using various antibodies at the following dilutions: WISP1 antibody (1:1000), β-catenin antibody (1:1000), Flag-tag antibody (1:1000), HA-tag antibody (1:1000) and normal rabbit/mouse IgG (Cell Signaling Technology).

### Growth of cells in athymic nude mouse and tumor size determination

Care of the laboratory animals and animal experimentation were performed in accordance with animal ethics guidelines and approved protocols. All animal studies were approved by the Animal Ethics Committee of the Fudan University Shanghai Cancer Center. LOVO cells infected with pLVX-AcGFP-C1-scramble shRNA (shNC) lentiviral vector or pLVX-AcGFP-C1-shWISP1 (shWISP1) were trypsinized and were washed and re-suspended in DMEM without FBS. Cell concentration and viability were determined using trypan blue. 12 male athymic nude mice were randomly divided into 2 groups (6 mice/group), and were subcutaneously injected by 2 × 10^6^ cells infected with shNC or shWISP1, respectively. The tumor size was determined every 3–4 days after tumor formed (around 1–2 weeks) as previously described [46]. Tumor volume was measured and calculated using the following formula: V (mm^3^) = 0.5 × larger diameter × smaller diameter^2^. 45 days later, the mice were sacrificed and photographed, and the tumors were weighted on a digital balance.

### Histology

Tissues sections were dehydrated and embedded in paraffin, and 2–3 μm sections were deparaffinized and stained sequentially with haematoxylin and eosin (H&E, Richard-Allan Scientific, Kalamazoo, MI). Stained tissue sections on slides were analyzed under identical light microscope (Axio Imager M1, Karl Zeiss, Germany) at ×200 magnifications.

### Immunohistochemistry

Tissues were initial treatment for deparaffinization and hydration and then heated in EDTA (pH 8.0) and incubated with 3% hydrogen peroxide for 10 min for antigen retrieval. The reaction of PCNA mouse monoclonal antibody (Abcam) was taken place 1 h at room temperature, following incubated by goat anti-mouse horseradish peroxidase-conjugated IgG (Abcam). Slides were stained with DAB (Shanghai Long Island Biotec. Co., LTD, China) and hematoxylin staining (Baso Diagnostics Inc., Zhuhai, China). Immunohistochemical signals were calculated with the positive staining cells. Stained tissue sections on slides were analyzed under identical light microscope at ×200 magnifications.

### Statistical analysis

Experimental data were presented as mean ± SD of at least three independent replicates through analyzing with GraphPad Prism 5. The two-tailed Student's *t*-test and One-way ANOVA analysis were used to assess comparisons between different groups. Overall survival in relation to WISP1 expression was evaluated by the Kaplan-Meyer survival curve and log-rank nonparametric test. Differences were considered significant at values of *P* < 0.05.
